# Draft Genome Sequences of *Staphylococcus aureus* AMRF1 (ST22) and AMRF2 (ST672), Ocular Methicillin-Resistant Isolates

**DOI:** 10.1128/genomeA.00168-14

**Published:** 2014-03-20

**Authors:** Nithya Velusamy, Logambiga Prakash, Neelamegam Sivakumar, Aju Antony, Lalitha Prajna, Vidyarani Mohankumar, Bharanidharan Devarajan

**Affiliations:** a Department of Ocular Microbiology, Aravind Medical Research Foundation, Madurai, India; b Department of Bioinformatics, Aravind Medical Research Foundation, Madurai, India; c BioSciences Core Lab, King Abdullah University of Science and Technology, Thuwal, Saudi Arabia

## Abstract

Sequence type 22 (ST22) and ST672 are the two major emerging clones of community-acquired methicillin-resistant *Staphylococcus aureus* in India. ST672 strains were found to cause severe ocular infections. We report the draft genome sequences of two emerging strains of methicillin-resistant *S. aureus*, AMRF1 (ST22) and AMRF2 (ST672), isolated from patients with ocular infections.

## GENOME ANNOUNCEMENT

*Staphylococcus aureus* is a common ophthalmic pathogen (1–5). In recent times, methicillin-resistant *S. aureus* (MRSA) clones, sequence type 772 (ST772) and ST22, have been reported in Indian communities (2). Studies have shown an increase in the pervasiveness of ocular MRSA infections (3, 4, 6). In India, ST772 continues to be responsible for more than half of ocular infections (2). The ST22 clone has been found in healthy young adults without any risk factors (7, 8). ST672, an emerging disease clone in India, harbors a type E immune evasion cluster and *tst1* genes (9, 10).

We announce the draft genome sequences of the methicillin-resistant *S. aureus* AMRF1 (ST22) and AMRF2 (ST672) strains. Strain AMRF1, isolated from a keratitis patient, belongs to staphylococcal cassette chromosome *mec* element (SCC*mec*) type IV and was positive for the Panton-Valentine leukocidin (PVL) gene. Strain AMRF2 was isolated from a patient with lower-lid suture infiltration, in which the infection was prolonged with fungal coinfection. This strain was also found to be PVL positive.

Whole-genome sequencing was performed using the Ion Torrent (PGM) sequencer with 400-bp read chemistry (Life Technologies). Sequencing was carried out as per the Ion 318 Chip sequencing protocol. The data were filtered with a Phred score of >20 to obtain ~32 million and ~28 million reads for the AMRF1 and AMRF2 strains, respectively, with an average coverage of >220×. The average read quality of 35 and minimum base quality of 30 were set to filter the reads for further analysis. Since a maximum coverage of 40× to 50× might compromise the quality of assemblies produced by the software MIRA version 4, we randomly subsampled 30% of the reads from these data. The assembly was performed on three sets of independently sampled read sets to ensure consistency.

The AMRF1 genome consists of 112, 101, and 99 contigs from three data sets, and the AMRF2 genome consists of 69, 60, and 75 contigs; the two strains cover a total length of ~2.96 and ~2.92 million bp, respectively, evidencing an almost ~0.04 Mb difference between two isolates. For both isolates, the average G+C content was estimated to be 32%. The Rapid Annotations using Subsystems Technology (RAST) version 4.0 server was used to annotate the draft genomes, and the results were amalgamated to yield maximum numbers. RAST provided the first clues in a comparative genomics analysis of the two clinical isolates. While 3,170 protein-coding sequences were revealed for AMRF1, AMRF2 has 3,120 protein-coding sequences. More than 2,848 protein-coding sequences were similar in the two isolates. However, we identified three copies of transposon 554 (Tn*554*)-related transposases A, B, and C, and phages only for ST22. In the case of ST672, genes encoding the fosfomycin resistance protein FosB and cadmium resistance protein were present as unique genes. Further comparative genomics analysis of these two strains and genome comparison with other MRSA strains will provide more valuable information on their virulence mechanisms.

### Nucleotide sequence accession numbers.

This whole-genome shotgun project has been deposited at DDBJ/EMBL/GenBank under the accession no. AZTC00000000 (*S. aureus* AMRF1) and JASM00000000 (*S. aureus* AMRF2). The versions described in this paper are the first versions, AZTC01000000 and JASM01000000.
